# Clinical Impact of PSMA PET Fusion-Based RT Planning on Dosimetry and PSA Response in Prostate Cancer

**DOI:** 10.3390/jcm15093394

**Published:** 2026-04-29

**Authors:** Sema Yilmaz Rakici, Sibel Goksel, Esra Aydın

**Affiliations:** 1 Department of Radiation Oncology, Faculty of Medicine, Recep Tayyip Erdogan University, 53100 Rize, Turkey; 2 Department of Nuclear Medicine, Faculty of Medicine, Adnan Menderes University, 09010 Aydin, Turkey; sibelkandemirgoksel@gmail.com; 3 Department of Medical Oncology, Faculty of Medicine, Recep Tayyip Erdogan University, 53100 Rize, Turkey; esra.aydin@erdogan.edu.tr

**Keywords:** prostate cancer, PSMA PET, multiparametric prostate MRI, RT planning, intraprostatic focal boost

## Abstract

**Background**: This study aimed to assess the impact of PSMA-PET/CT fusion imaging on target volume delineation in prostate cancer RT and to evaluate its effects on dosimetric parameters and PSA response, including intraprostatic boost. **Methods**: This single-center, retrospective study included 138 prostate cancer patients treated with definitive RT. Patients were evaluated according to the use of PSMA-PET/CT fusion-based planning and an intraprostatic focal boost, and dosimetric parameters for target volumes and organs at risk were compared. **Results**: PSMA-PET/CT fusion-based planning significantly increased the minimum dose coverage of the prostate target volume (96.7% vs. 95.5%, *p* = 0.003) while reducing the maximum dose (104.8% vs. 106.1%, *p* < 0.001). At 1 year after RT, the median change in PSA from baseline was 0.08 ng/mL (range, −0.44–2.12) in patients who underwent PSMA PET imaging-based fusion planning compared with 0.01 ng/mL (range, −0.049–4.07) in those who did not (*p* = 0.010). In patients receiving the intraprostatic focal boost with PSMA-PET/CT fusion, rectal maximum dose percentages were significantly lower than in those without the boost (103.2% [98.9–106.7] vs. 103.8% [95.7–107.4], *p* = 0.026). Rectal V65 and V50 values were also significantly reduced in the fusion group (7.0% [0.7–19.8] vs. 5.2% [1.2–21.7], *p* = 0.007; and 13.6% [6.3–21.9] vs. 11.4% [4.4–29.2], *p* = 0.027, respectively). Bladder maximum dose percentages were significantly lower in patients receiving the PSMA-PET/CT fusion-guided intraprostatic boost compared with those without the boost (102.6% [99.7–107.9] vs. 104.5% [99.1–108.5], *p* = 0.001). **Conclusions**: PSMA-PET/CT fusion-based planning improves biologically guided target delineation and dose homogeneity and suggests potential for better early biochemical response while reducing normal tissue exposure, whereas the intraprostatic focal boost improves dose distribution but is not associated with a significant short-term (1-year) PSA benefit.

## 1. Introduction

Prostate cancer is one of the most common solid malignancies in men and remains a leading cause of cancer-related morbidity and mortality worldwide. Alongside surgery and androgen deprivation therapy, radiotherapy (RT) plays a central role as a definitive treatment modality across all risk groups, providing durable long-term disease control [1]. Advances in modern imaging and RT techniques have substantially improved treatment efficacy and safety by enhancing target volume accuracy and minimizing radiation exposure to surrounding normal tissues [2]. High-precision prostate RT improves in-field tumor control while reducing treatment-related toxicity. Currently, computed tomography (CT) and magnetic resonance imaging (MRI) are widely used for target volume delineation in prostate RT planning [3]. MRI offers superior soft-tissue contrast, multiparametric imaging capabilities, and the advantage of avoiding ionizing radiation compared with CT. However, both CT and MRI primarily provide anatomical information and offer limited insight into the biological behavior and intraprostatic heterogeneity of prostate tumors. The introduction of positron emission tomography (PET) targeting prostate-specific membrane antigen (PSMA) has addressed this limitation. PSMA ligand PET using 68Ga-labeled tracers targets a type II transmembrane glycoprotein that is overexpressed at levels 100–1000 times higher in prostate cancer cells compared with normal tissues, making it a highly sensitive and specific biomarker for prostate cancer imaging [4]. Several studies have demonstrated that PSMA-targeted PET is superior to conventional imaging modalities, including CT, MRI, and 18F-FDG PET, for the detection of primary and metastatic prostate cancer lesions. Moreover, PSMA PET has shown complementary and, in certain clinical settings, superior diagnostic performance compared with prostate multiparametric magnetic resonance imaging (mpMRI) in prostate cancer staging and lesion localization [5,6,7].

Despite its growing role in diagnosis and staging, robust clinical data regarding the integration of PSMA PET into RT planning, particularly through fusion with planning CT for target delineation, remain limited. In prostate cancer patients undergoing RT, PSMA PET and mpMRI-based target definition may represent an effective imaging approach for defining the clinical target volume (CTV) and gross tumor volume (GTV), especially in the context of increasingly personalized RT strategies. The incorporation of PSMA PET into RT planning has the potential to guide biologically informed target delineation, optimize dose distribution, and support individualized treatment adaptation.

The aim of this study was to evaluate the contribution of PSMA PET-based target volume delineation in RT planning for prostate cancer and to assess its added value compared with conventional imaging-based approaches. Unlike previous studies that primarily focused on the diagnostic or staging performance of PSMA PET imaging, the present study specifically investigates its clinical impact on RT planning by integrating PSMA PET/CT fusion into target volume delineation. This work evaluates not only dosimetric improvements associated with a biologically guided intraprostatic focal boost but also correlates these changes with early prostate-specific antigen (PSA) response. By simultaneously analyzing target coverage, organ-at-risk sparing, and biochemical outcomes in a contemporary cohort treated with hypofractionated volumetric modulated arc therapy (VMAT), this study provides clinically relevant evidence supporting the role of PSMA PET-based planning as a biologically informed strategy in personalized prostate RT.

## 2. Materials and Methods 

External beam radiotherapy was delivered using a Varian linear accelerator (Trilogy, Varian Medical Systems, Palo Alto, CA, USA). Treatment planning, encompassing image fusion and dosimetric evaluation, was performed via the Eclipse™ Treatment Planning System (v13.6; Varian Medical Systems). Simulation and planning CT datasets were acquired using an Aquilion™ LB CT simulator (Toshiba Medical Systems, Tokyo, Japan). Ga-PSMA PET/CT images were obtained using a Biograph™ mCT 20 Excel PET/CT scanner (Siemens Healthcare GmbH, Erlangen, Germany).

### 2.1. Patient Selection and Study Design

This retrospective study included patients with prostate cancer who underwent definitive external beam radiotherapy at our institution between January 2022 and May 2025. The study protocol was approved by the institutional ethics committee, and the initial study period was subsequently extended following an additional ethics committee amendment to allow the inclusion of newly eligible patients. Written informed consent for participation in the study and for the use of clinical and imaging data for research purposes was obtained from all patients prior to treatment. All procedures performed in this study were conducted in accordance with the ethical standards of the institutional research committee and with the Declaration of Helsinki.

A total of 138 patients (*n* = 138) were included in the final statistical analysis. None of the patients had received prior treatment for prostate cancer, and no patient had contraindications to MRI or PSMA PET/CT. Baseline clinical characteristics, including age, TNM (tumor–node–metastasis) stage, Gleason score, PSA level, and pathological findings from biopsy specimens, were retrieved from institutional medical records. TNM classification was used for clinical staging (cTNM). Prostate mpMRI was performed in 107 patients, while PSMA PET/CT imaging was available for 95 patients.

### 2.2. Data Collection

Clinical data including diagnosis, TNM stage, Gleason score, PSA level, and presence of perineural invasion (PNI) were obtained from the hospital information system. RT plans were reviewed using the treatment planning system (TPS), and dosimetric parameters for target volumes (prostate, seminal vesicles, and pelvic lymph node regions) as well as organs at risk (OARs), including the bladder, rectum, femoral heads, and whole body, were recorded. Plans incorporating PSMA PET/CT fusion were compared with those without fusion, and PSA response was analyzed accordingly.

### 2.3. Imaging and Fusion Procedure

PSMA PET/CT images were fused with planning CT images within the treatment planning system (TPS) to integrate anatomical, functional, and metabolic information for radiotherapy planning. All PSMA PET/CT scans were acquired using an RT flat tabletop to ensure consistent patient positioning and to improve anatomical correspondence with the planning CT, thereby enhancing fusion accuracy. Image registration between PSMA PET/CT and planning CT was performed using rigid fusion within the TPS. No deformable registration was applied. Fusion accuracy was visually verified based on anatomical landmarks.

**Figure 1:**
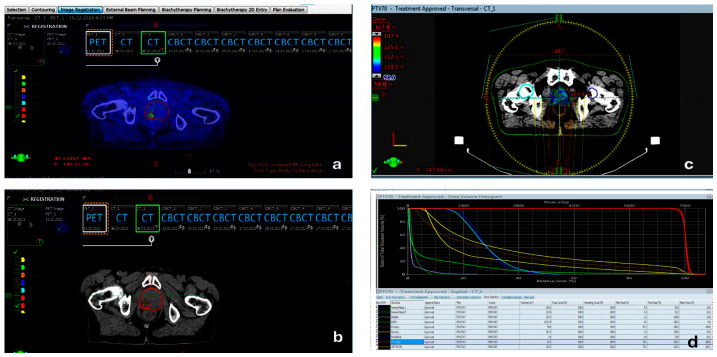
Representative case illustrating PSMA PET/CT-guided radiotherapy planning. (**a**) PSMA PET images demonstrating focal intraprostatic tracer uptake corresponding to the dominant lesion (SUVmax-based visual assessment); (**b**) corresponding radiotherapy planning CT acquired with an RT flat tabletop; (**c**) fused PSMA PET/CT images with delineated target volumes, including gross tumor volume (GTV) and clinical target volume (CTV), along with organs at risk (OARs), following rigid image registration within the treatment planning system; (**d**) three-dimensional radiotherapy dose distribution and associated dose–volume histograms (DVHs) illustrating target coverage and OAR sparing, including the intraprostatic focal boost applied to the PSMA-avid lesion. Target volumes are displayed in red, while organs at risk are shown in different colors according to the default color scheme of the treatment planning system.

Intraprostatic tumor foci were delineated as gross tumor volumes (GTVs) based on visually identified focal PSMA uptake corresponding to SUVmax, without applying a fixed absolute SUV threshold or a predefined percentage of SUVmax. Instead, focal areas of increased tracer uptake consistent with dominant intraprostatic lesions were contoured directly on the fused PSMA PET/CT images, as demonstrated in Figure 1. This approach is consistent with previously published PSMA PET-guided radiotherapy planning methodologies.

All GTV delineations were performed by a single experienced radiation oncologist to minimize interobserver variability, which is now explicitly stated. In patients with pelvic lymph node metastases, PSMA-avid lymph nodes were contoured as nodal GTVs, and the treatment field was expanded to include the pelvic lymphatic regions in accordance with RTOG protocols. The clinical target volume (CTV) included the entire prostate and, in high-risk patients, the proximal 1–2.5 cm of the seminal vesicles, following established RTOG guidelines. In cases with nodal involvement, the pelvic lymphatic CTV was defined according to anatomical regions described in the SPPORT trial. Patients without lymph node metastases received prostate-only irradiation. GTVs were expanded isotropically by 3–5 mm to generate the planning target volume (PTV), consistent with RTOG recommendations. Figure 1 illustrates PSMA PET/CT-to-planning CT fusion, intraprostatic GTV delineation based on focal PSMA uptake, and the corresponding dose distribution in a representative patient.

### 2.4. GTV, CTV, PTV, and Dose Definitions

GTVs were delineated based on visually identified focal PSMA uptake corresponding to SUVmax on PSMA PET/CT images. All contours were performed by a single experienced radiation oncologist using rigid PET/CT-to-planning CT registration within the treatment planning system [7,8]. Figure 1 demonstrates PSMA PET/CT fusion with planning CT, GTV delineation, and corresponding dose distributions in a representative patient. In patients with pelvic lymph node metastases, the treatment field was expanded to include pelvic lymphatic regions in accordance with the RTOG 0924 protocol, and nodal GTVs were defined for involved lymph nodes [9]. The CTV was delineated to include the entire prostate and, in high-risk patients, the proximal 1–2.5 cm of the seminal vesicles, following the RTOG 0126 and RTOG 0415 guidelines [10,11]. In cases with nodal involvement, the pelvic lymphatic CTV was defined according to the anatomical regions described in the RTOG 0534 (SPPORT) trial [9]. Patients without lymph node metastases received prostate-only irradiation. All GTV delineations were performed by an experienced radiation oncologist based on SUVmax uptake on PET/CT. GTVs were expanded isotropically by 3–5 mm to generate the planning target volume (PTV), consistent with the RTOG 0415 protocol [12]. A summary of treatment fields and prescribed dose levels is provided in the corresponding table.

### 2.5. Dosimetric Analysis

Dosimetric evaluation was systematically performed using dose–volume histograms (DVHs) for target volumes (prostate, seminal vesicles, and pelvic lymph nodes) and organs at risk (rectum, bladder, femoral heads, penile bulb, and whole body). Minimum dose (Dmin), mean dose (Dmean), and maximum dose (Dmax) values were recorded. To ensure comparability across different fractionation schedules and prescribed doses, all dosimetric parameters were normalized to the prescribed dose and expressed as percentages (%).

### 2.6. Ethics Approval and Patient Consent

This study was designed as a retrospective study using routinely collected clinical data obtained from hospital archives. Written informed consent for the use of medical data for scientific and research purposes was obtained from all patients admitted to the Department of Radiation Oncology prior to treatment, and these consent forms are available in the patients’ medical records.

Permission to use hospital archive data and patient medical information was obtained from the relevant Provincial Health Directorate (Approval No: E-64960800-799-214610600; date: 2 May 2023). In addition, the study was approved by the institutional ethics committee (Approval No.: E-40465587-050.01.04-729, dated 7 June 2023; decision no. 2023/144). After the initial approval, the study period was extended through a subsequent ethics committee amendment to allow the inclusion of additional eligible patients. Accordingly, patients treated and followed until May 2025 were included in the final analysis. The study was conducted in accordance with the ethical principles of the Declaration of Helsinki (1975, revised in 2013). In compliance with Article 23 of the Declaration of Helsinki, approval was obtained from the local institutional review board/ethics committee prior to the initiation of the study, and the research was carried out in accordance with both national and international ethical guidelines. Written informed consent for the scientific use of medical data was obtained from all patients, and all data were anonymized prior to analysis. No identifiable patient information or images are presented in this article.

### 2.7. Statistical Analysis

A total of 138 patients (*n* = 138) were included in the final statistical analysis. Data entry and statistical analyses were performed using SPSS for Windows, version 18.0 (SPSS Inc., Chicago, IL, USA). The normality of continuous variables was assessed using visual methods (histograms and probability plots) and analytical tests (Kolmogorov–Smirnov and Shapiro–Wilk tests). As numerical data were not normally distributed, descriptive statistics were reported as median, minimum, and maximum values, while categorical variables were presented as frequencies and percentages.

Non-parametric tests were used for statistical comparisons. The Mann–Whitney U test was applied for comparisons between two independent groups, while the Kruskal–Wallis test was used for comparisons among three or more groups. When the Kruskal–Wallis test indicated statistical significance, pairwise comparisons were performed using the Mann–Whitney U test with Bonferroni correction for multiple comparisons. A *p* value < 0.05 was considered statistically significant.

## 3. Results

**Figure 2:**
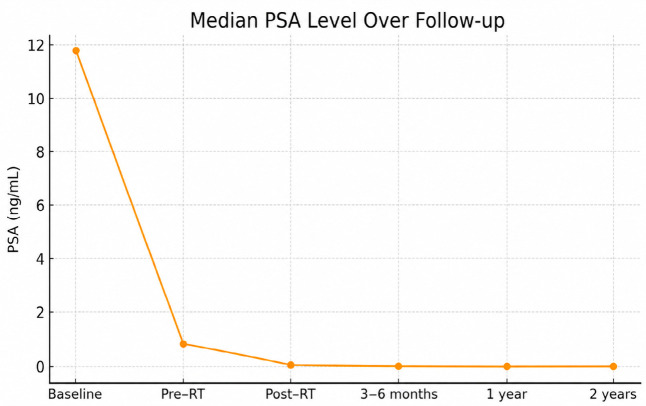
Median prostate-specific antigen (PSA) levels at baseline, prior to RT, immediately after RT, and during follow-up, demonstrating a marked decline following treatment.

The median age of the study population was 70.0 years (range: 44–84 years). The median follow-up duration was 20.15 months (range: 0.76–109.21 months). The median baseline PSA level was 11.85 ng/mL and decreased to 0.94 ng/mL prior to RT, reflecting the effect of neoadjuvant androgen deprivation therapy (ADT), when administered. After completion of RT, the median PSA level declined to 0.15 ng/mL, further decreasing to 0.07 ng/mL at 3–6 months of follow-up, 0.04 ng/mL at 1 year, and 0.11 ng/mL at 2 years. The temporal change in PSA levels is illustrated in Figure 2.

The median Gleason score was 7 (range: 6–9). Perineural invasion (PNI) was present in 39.6% of patients (*n* = 53) and absent in 60.4% (*n* = 81). According to pathological T staging, the most common stage was T2c (42.0%, *n* = 58), followed by T2a (28.3%, *n* = 39).

Among the 138 patients included in the study, mpMRI was performed in 107 patients, while PSMA PET/CT imaging was available in 95 patients. The most frequently observed Prostate Imaging-Reporting and Data System (PI-RADS) score on mpMRI was score 5 (50.5%, *n* = 54), followed by score 4 (27.1%, *n* = 29). Based on MRI findings, the most common clinical T stage was cT2a (36.4%, *n* = 39). Seminal vesicle invasion was detected in 16.7% of patients (*n* = 18), and metastatic pelvic lymph node involvement was identified in 11.1% (*n* = 12).

In patients who underwent PSMA PET/CT imaging, the most frequent T stage was cT2a (23.2%, *n* = 22). Seminal vesicle involvement was observed in 12.6% (*n* = 12), pelvic lymph node metastases in 22.1% (*n* = 21), and distant metastases in 5.3% of patients (*n* = 5). Detailed demographic, clinical, and radiological characteristics of the study cohort are summarized in Table 1.

### Dosimetric Outcomes: Target Volume Coverage, PSMA PET/CT Fusion-Guided Intraprostatic Boost, and PSA Response

Table 2 summarizes the associations between RT planning-related factors and PSA changes from post-RT to 1-year follow-up, together with minimum, maximum, and mean prostate dose distributions. Dosimetric data related to the target volume coverage, PSMA PET/CT fusion-guided intraprostatic boost, and PSA response are summarized in Table 2. Among the study cohort, 68.8% of patients (*n* = 95) underwent PSMA PET/CT imaging, which was fused with planning CT for RT planning. Based on PSMA PET findings, 44 patients (31.9%) received an intraprostatic focal boost.

In patients planned with PSMA PET/CT fusion, the minimum dose covering the prostate target volume was significantly higher compared with those without PET fusion (median 96.7% [64.1–99.1] vs. 95.5% [66.8–98.5], *p* = 0.003), while the maximum dose was significantly lower (104.8% [81.5–108.8] vs. 106.1% [102.4–108.1], *p* < 0.001). No significant difference was observed in the mean prostate dose between the two groups (*p* = 0.142). These findings indicate a more homogeneous dose distribution within the prostate target volume when PSMA PET/CT fusion-based planning was employed.

When patients were stratified by fractionation scheme, no significant difference in PSA reduction was observed between conventionally fractionated RT (2 Gy per fraction; 52.9%, *n* = 73) and hypofractionated regimens (2.5 Gy per fraction; 47.1%, *n* = 65) (*p* = 0.186). Similarly, the intraprostatic focal boost did not result in a statistically significant difference in PSA change at 1 year compared with plans without the boost (median PSA change: 0.06 ng/mL in both groups, *p* = 0.565).

RT technique analysis showed that VMAT was used in the majority of patients (79.0%). While PSA reduction did not differ between VMAT and intensity-modulated radiation therapy (IMRT) techniques (*p* = 0.681), the maximum dose to the prostate target volume was significantly higher in VMAT plans compared with IMRT (105.5% [81.5–108.8] vs. 104.8% [83.3–108.7], *p* = 0.020).

Comparison of RT target volumes (prostate-only, prostate plus seminal vesicle, and pelvic fields) revealed no significant differences in PSA reduction or prostate dose parameters (*p* > 0.05). Likewise, total prescribed dose levels (70, 72.5, 74, 76, 78, and 80 Gy) were not associated with differences in PSA reduction (*p* = 0.687). However, significant differences were observed among total dose groups with respect to minimum, maximum, and mean prostate doses (*p* = 0.016, *p* < 0.001, and *p* = 0.002, respectively). Post hoc analysis demonstrated that the 70 Gy group had a higher minimum prostate dose compared with the 76 Gy and 80 Gy groups (Table 2).

Prostate volume categories (<50 cc, 50–70 cc, 70–100 cc, and >100 cc) did not significantly affect PSA reduction or prostate dosimetric parameters (*p* > 0.05).

Patients treated with PSMA PET/CT fusion-based planning demonstrated a significantly greater PSA decline at 1 year compared with those without PET fusion (median PSA change: 0.08 ng/mL [−0.44–2.12] vs. 0.01 ng/mL [−0.049–4.07], *p* = 0.010). In contrast, the intraprostatic focal boost did not confer an additional PSA benefit at 1 year (*p* = 0.565) (Table 2).

Rectal dosimetric outcomes are summarized in Table 3. In patients receiving the intraprostatic focal boost, the rectal maximum dose was significantly lower than in those without the boost (103.2% [98.9–106.7] vs. 103.8% [95.7–107.4], *p* = 0.026). Rectal V65 and V50 values did not differ significantly according to the use of an intraprostatic boost. However, patients planned with PSMA PET/CT fusion-based radiotherapy demonstrated significantly lower rectal dose exposure compared with those planned without PSMA PET/CT fusion, with reduced rectal V65 (7.0% [0.7–19.8] vs. 5.2% [1.2–21.7], *p* = 0.007) and rectal V50 (13.6% [6.3–21.9] vs. 11.4% [4.4–29.2], *p* = 0.027).

Hypofractionated RT plans were associated with lower rectal dose parameters compared with conventionally fractionated plans. VMAT plans resulted in significantly lower rectal minimum and mean doses compared with IMRT (*p* < 0.05). Plans including pelvic irradiation demonstrated significantly higher rectal minimum and mean doses compared with prostate-only or prostate plus seminal vesicle fields (post hoc: pelvis > prostate, SV; *p* < 0.001). Increasing total prescribed dose (≥78 Gy) was associated with significantly higher rectal dose parameters, with post hoc analysis showing that 78 Gy and 80 Gy plans delivered higher rectal doses than 70–72.5 Gy plans (Table 3).

Bladder dosimetric parameters were not significantly affected by PSMA PET/CT fusion alone; however, patients receiving the PSMA PET-guided intraprostatic focal boost exhibited a significantly lower bladder maximum dose compared with those without the boost (102.6% [99.7–107.9] vs. 104.5% [99.1–108.5], *p* = 0.001). VMAT plans yielded significantly lower bladder doses than IMRT (*p* < 0.05). Pelvic irradiation was associated with significantly higher bladder minimum, maximum, and mean doses compared with prostate-only or prostate plus seminal vesicle plans, with post hoc analysis confirming consistently higher OAR doses in pelvic fields. Higher total prescribed doses (≥78 Gy) were associated with increased bladder minimum and mean doses (*p* < 0.05) (Table 3).

Finally, dosimetric analysis revealed that femoral heads, penile bulb, and whole-body dose parameters were significantly higher in patients treated without PSMA PET/CT fusion, without an intraprostatic boost, with conventional fractionation, and using the IMRT technique (Table 3). Pelvic irradiation and higher total dose levels (≥76 Gy) were associated with increased femoral head maximum doses and penile bulb mean doses. In contrast, hypofractionated regimens and PSMA PET-guided planning achieved improved sparing of femoral heads, penile bulb, and whole-body dose exposure.

## 4. Discussion

PSMA PET-guided RT planning improves target volume delineation accuracy, resulting in enhanced dose homogeneity while simultaneously reducing unnecessary radiation exposure to surrounding normal tissues. Sonni et al. [13] demonstrated that PSMA PET-based contouring provides a more precise target definition and may translate into clinical benefit. Furthermore, PSMA PET data have been shown to contribute to the optimization of focal boost strategies during post-therapy evaluations [14]. Previous studies have also reported that focal boost approaches delivered with VMAT or IMRT optimize organ-at-risk (OAR) sparing in prostate cancer RT [15].

Modern RT techniques incorporating MRI and PSMA PET/CT fusion—particularly VMAT and hypofractionated regimens—enable improved target coverage while achieving lower dose exposure to critical organs. Consistent with these reports, our findings support PSMA PET fusion-guided hypofractionated VMAT planning as a contemporary approach that enhances both target volume homogeneity and OAR protection.

Our findings further support the evolving paradigm of biologically guided radiotherapy in prostate cancer, in which advanced imaging modalities play a central role in improving treatment planning and early biochemical response. Previous evidence has demonstrated that multiparametric MRI reliably detects clinically significant prostate cancer not only in biopsy-naïve patients but also in those with prior negative biopsies, thereby reducing unnecessary repeat biopsies and strengthening confidence in imaging-based risk stratification [16]. In addition, MRI-derived prostate volume estimation has been shown to correlate well with surgical specimens while offering advantages in cost effectiveness and workflow efficiency, underscoring its clinical utility for treatment planning and follow-up [17]. Collectively, these data reinforce the clinical relevance of combining high-quality anatomical and molecular imaging to optimize radiotherapy delivery and support further prospective validation.

Multiple studies have demonstrated that PSMA PET has significantly higher sensitivity for detecting pelvic lymph node involvement compared with mpMRI. In a systematic review and meta-analysis by Yang et al. [18], PSMA PET demonstrated a sensitivity of 74% and a specificity of 96% for lymph node metastasis detection, markedly outperforming mpMRI. Similarly, Petersen et al. [19] reported superior diagnostic accuracy of PSMA PET/CT compared with MRI/CT (sensitivity 39% vs. 8%). Moreover, hybrid 68Ga-PSMA PET/MRI has been shown to allow for simultaneous evaluation of the primary tumor, metastatic lymph nodes, and bone metastases [20]. These data support our findings and underscore the complementary role of PSMA PET in treatment planning for nodal and distant disease assessment.

In a multicenter randomized phase III trial involving intermediate- and high-risk prostate cancer patients, external beam RT (EBRT) delivered either to the whole prostate (77 Gy in 35 fractions) or with a focal boost up to 95 Gy to MRI-visible lesions demonstrated significantly improved disease-free survival (DFS) in the focal boost arm without additional toxicity at 5 years, with sustained benefit observed at the 10-year follow-up [21].

In another study comparing 68Ga-PSMA PET/CT- and mpMRI-guided RT planning, boost volumes were similar in size, although spatial concordance was limited. Nevertheless, based on favorable acute toxicity profiles, the PSMA PET-guided intraprostatic boost was considered a safe strategy for dose escalation in prostate SBRT [7]. Dosimetric analyses by Goodman et al. [8] showed that PSMA PET-defined dominant intraprostatic lesion boosts resulted in target-specific dose escalation while maintaining overall plan homogeneity. Similarly, Singh et al. [7] demonstrated that PSMA PET-guided SBRT boost plans achieved high-dose conformity without significantly altering the mean prostate dose. Li et al. [22] further showed that integrating PSMA PET into adaptive planning optimized dose escalation, particularly in plans involving extended pelvic fields. Collectively, these findings are consistent with our results, supporting PSMA PET-guided boost planning as a strategy that enables targeted dose intensification while preserving homogeneity.

The combination of androgen deprivation therapy and RT has been shown to synergistically enhance PSA decline dynamics and improve long-term biochemical control by targeting micrometastatic disease [23]. Additionally, PSMA PET-guided boost strategies have been associated with more pronounced long-term PSA responses, further optimizing biochemical outcomes [22]. These observations suggest that the PSA decline patterns observed in this study may be indicative of a favorable treatment response and improved local disease control; however, PSA decline represents a surrogate biochemical marker and should be interpreted with caution, as it cannot be equated with durable disease control, survival outcomes, or long-term clinical toxicity, as is well recognized.

Lower rectal doses observed in hypofractionated regimens suggest that modern techniques can be more effectively implemented within these fractionation schedules [24]. VMAT, in particular, achieved significant reductions in rectal mean and minimum doses through improved dose conformality and modulation, thereby potentially lowering toxicity risk compared with IMRT. While increased rectal dose exposure in pelvic field plans is expected, plans limited to the prostate or prostate plus seminal vesicles demonstrated significantly lower rectal doses. Importantly, lower rectal V65 and V50 values in PSMA PET-guided plans indicate improved target delineation and enhanced OAR sparing, highlighting the dosimetric optimization potential of PSMA PET beyond tumor localization alone.

Boost application and fractionation schemes did not significantly affect bladder mean dose; however, IMRT plans were associated with higher minimum, maximum, and mean bladder doses compared with VMAT. This finding emphasizes the superiority of VMAT in bladder protection through enhanced dose conformality. As expected, inclusion of pelvic fields and higher total doses (≥78 Gy) resulted in increased bladder and rectal doses. Nevertheless, no significant differences in bladder dosimetry were observed between PSMA PET and non-PSMA PET plans, suggesting that PSMA PET primarily contributes to target accuracy rather than direct bladder sparing.

Higher femoral head, penile bulb, and body doses observed in patients treated without PSMA PET fusion, without the boost, with conventional fractionation, and using IMRT likely reflect the limitations of traditional planning approaches in accurately distinguishing anatomical and functional structures. Expanded pelvic volumes and high total doses (≥76 Gy) further contributed to increased dose spill to adjacent organs. In contrast, hypofractionated regimens combined with PSMA PET-guided planning and modern techniques enabled more precise tumor targeting, reduced target volumes, and substantially improved OAR sparing.

Recent studies have consistently shown that PSMA PET-guided RT planning, particularly when combined with VMAT, significantly reduces OAR doses. Bartlett et al. [25] demonstrated improved rectal and bladder sparing using partial-arc VMAT in pelvic prostate cancer RT. Calais et al. [26] emphasized that PSMA PET fusion enhances target delineation accuracy, thereby reducing dose to critical organs. Similarly, Thomas et al. [27] reported that intraprostatic boost using 68Ga-PSMA PET/CT-based IMRT maintained rectal and bladder doses within clinically acceptable limits. These data further support the role of PSMA PET-based planning in improving dose conformality and minimizing toxicity [28].

The primary strength of this study lies in its comprehensive dosimetric analysis of PSMA PET fusion-guided RT planning in a real-world clinical cohort, including evaluation of target volumes, intraprostatic boost strategies, PSA response, and multiple organs at risk. The inclusion of various planning techniques, fractionation schemes, and treatment volumes provides a broad perspective on contemporary RT practice. However, the retrospective design and single-institution nature of the study may limit generalizability. Additionally, relatively short follow-up restricts conclusions regarding long-term biochemical control and late toxicity. Prospective, multicenter studies with extended follow-up are warranted to further validate the clinical impact of PSMA PET-guided RT planning.

Recent studies highlight a paradigm shift in prostate cancer management toward multimodal strategies integrating serum biomarkers, advanced imaging, systemic therapies, and molecular profiling [29]. The clinical value of PSA is increasingly enhanced when interpreted alongside imaging modalities such as biparametric MRI, which has been shown to improve risk stratification and reduce unnecessary biopsies in patients with elevated PSA levels [30]. Beyond imaging, molecular studies have identified key drivers of treatment response and resistance, including immune-related and chemotherapy-associated biomarkers such as ITGA2, as well as aneuploidy driver genes linked to tumor progression, immune modulation, and therapeutic vulnerability [31]. Furthermore, integrated multi-omics approaches have demonstrated that immune heterogeneity, genomic instability, and metabolic dependencies collectively shape drug resistance and prognosis in prostate cancer [32]. Within this evolving framework, our findings on PSA response and treatment-related adverse effects following PSMA PET fusion-guided interventions support the translational relevance of combining advanced molecular imaging with PSA dynamics to better predict treatment response and inform personalized therapeutic strategies.

Although PSMA PET/CT imaging entails additional upfront costs and is not uniformly recommended for routine use across all prostate cancer risk groups, accumulating evidence supports its cost-effective clinical value, particularly in intermediate- and high-risk disease. Multiple economic evaluations have demonstrated that PSMA PET/CT offers superior staging accuracy compared with conventional imaging, especially for the detection of occult nodal and distant metastases, leading to more appropriate treatment selection and potential reductions in downstream costs associated with mis-staging and unnecessary interventions [33,34]. From a radiotherapy perspective, PSMA PET/CT-guided planning enables biologically informed and anatomically precise target volume delineation, addressing a major limitation of traditional pelvic lymph node irradiation strategies that rely on formula-based risk estimates rather than direct visualization. This individualized approach may reduce overtreatment of uninvolved nodal regions while improving dose conformity and sparing of organs at risk. Cost-effectiveness analyses further suggest that despite higher imaging costs, PSMA PET/CT can be economically justified by improving quality-adjusted life years (QALYs) and optimizing treatment pathways, particularly in high-risk patients [35,36]. Nevertheless, the incremental benefit appears more modest in low-risk and selected intermediate-risk populations, underscoring the need for prospective trials and health-economic studies to better define patient subgroups that derive the greatest clinical and economic benefits and to establish the optimal integration of PSMA PET/CT into routine radiotherapy workflows.

Interpretation of PSA dynamics should be approached with caution, as pre-radiotherapy PSA levels may reflect the effects of neoadjuvant androgen deprivation therapy where administered. In addition, several clinically relevant variables—including disease risk stratification, duration of androgen deprivation therapy, and the use of multiparametric MRI—were not uniformly controlled across this heterogeneous cohort, thereby introducing the potential for residual confounding. The retrospective, single-center design of the study represents an inherent limitation, along with substantial heterogeneity in disease stage, radiotherapy dose and fractionation schedules, treatment volumes, imaging availability, and androgen deprivation therapy utilization. Furthermore, the non-randomized allocation of PSMA PET/CT imaging and intraprostatic boosts may have introduced selection bias, and the possibility of unmeasured confounding cannot be fully excluded. Another important limitation is the absence of clinical toxicity data; therefore, the dosimetric improvements observed in this study cannot be directly extrapolated to reductions in patient-reported or clinician-assessed toxicity.

## 5. Conclusions

This study demonstrates that PSMA PET/CT fusion enhances radiotherapy planning accuracy in prostate cancer, resulting in improved target volume dose homogeneity. The observed increase in the minimum dose and reduction in the maximum dose within the prostate reflect improved dose uniformity in PSMA PET-guided plans. Moreover, the greater PSA decline observed at one year in patients planned with PSMA PET/CT suggests a potential association between biologically targeted delineation and improved early biochemical response.

While the intraprostatic focal boost achieved targeted dose escalation, it did not result in a short-term PSA advantage; however, it may offer potential benefits for long-term biochemical control. Among modern RT techniques, VMAT provided superior rectal and bladder sparing compared with IMRT, and hypofractionated regimens further reduced rectal mean and minimum doses, thereby lowering toxicity risk. Reduced rectal V65 and V50 values in PSMA PET fusion plans confirm the contribution of improved target delineation to critical organ protection.

As expected, inclusion of pelvic treatment fields was associated with increased rectal and bladder doses due to expanded target volumes. Importantly, the combined use of PSMA PET and modern planning approaches resulted in significantly lower doses to the femoral heads, penile bulb, and body, emphasizing their organ-sparing advantage. Overall, these findings support PSMA PET fusion-guided hypofractionated VMAT planning as a safe and effective RT strategy that delivers highly accurate target coverage while minimizing radiation exposure to surrounding normal tissues. These findings support the growing role of PSMA PET-guided planning as a promising component of biologically guided radiotherapy in prostate cancer, underscoring the paradigm shift from purely anatomical to metabolically informed treatment planning and providing a rationale for future prospective, randomized, and multicenter studies to validate long-term clinical benefits and toxicity profiles.

## Figures and Tables

**Table 1:** Demographic, clinical, and radiological characteristics of the study cohort.

Continuous Variables		Value
Age (years)		70.0 (44.0–84.0)
Follow-up duration (months)		20.15 (0.76–109.21)
Baseline PSA (ng/mL)		11.85 (0.04–99.00)
Gleason score		7 (6–9)
PNI (*n* = 134)		53 (39.6%)
Pathologic T Stage	T1c	5 (3.6%)
	T2a	39 (28.3%)
	T2b	27 (19.6%)
	T2c	58 (42.0%)
	T3a	9 (6.5%)
PI-RADS Score (*n* = 107)	Unspecified	7 (6.5%)
	1	1 (0.9%)
	2	6 (5.6%)
	3	10 (9.3%)
	4	29 (27.1%)
	5	54 (50.5%)
MRI T Stage (*n* = 107)	Unspecified	8 (7.5%)
	T2a	39 (36.4%)
	T2b	14 (13.1%)
	T2c	17 (15.9%)
	T3a	10 (9.3%)
	T3b	12 (11.2%)
	T4a	7 (6.5%)
MRI Seminal Vesicle Invasion (*n* = 107)	Absent	89 (83.2%)
	Present	18 (16.8%)
MRI Lymph Node Involvement (*n* = 107)	Absent	95 (88.8%)
	Present	12 (11.2%)
PSMA PET/CT Lymph Node (*n* = 95)	Absent	74 (77.9%)
	Present	21 (22.1%)
PSMA PET/CT Tumor Stage (*n* = 95)	T2a	22 (23.2%)
	T2b	16 (16.8%)
	T2c	21 (22.1%)
	T3a	13 (13.7%)
	T3b	12 (12.6%)
	T4a	5 (5.3%)
PSMA PET/CT Distal Metastasis (*n* = 95)	Absent	90 (94.7%)
	Present	5 (5.3%)

**Table 2:** Association of RT planning parameters with PSA decline and prostate dose distribution.

Characteristics		*n* (%)	PSA Change at 1 Year Post-RT	Prostate-Encompassing RT Dose (Gy)		
				Dmin	Dmax	Dmean
			Median (Min–Max)	Median (Min–Max)	Median (Min–Max)	Median (Min–Max)
PSMA PET/CT Imaging	No	43 (31.2)	0.01 (−0.049–4.07)	95.5 (66.8–98.5)	106.1 (102.4–108.1)	101.0 (97.8–103.4)
	Yes	95 (68.8)	0.08 (−0.44–2.12)	96.7 (64.1–99.1)	104.8 (81.5–108.8)	100.9 (77.7–105.5)
	*p* value *		**0.010**	**0.003**	**<0.001**	0.142
Intraprostatic Focal Boost	No	94 (68.1)	0.06 (−0.49–4.07)	96.6 (66.8–99.1)	105.8 (81.5–108.8)	101.1 (77.7–103.4)
	Yes	44 (31.9)	0.06 (0.05–1.76)	95.6 (64.1–98.2)	104.0 (83.3–106.8)	100.3 (78.9–105.5)
	*p* value *		0.565	0.178	**<0.001**	**<0.001**
Fractionation	Conv	73 (52.9)	0.05 (−0.49–4.0)	96.1 (66.8–99.1)	105.8 (81.5–108.8)	101.0 (77.7–103.4)
	Hypo	65 (47.1)	0.07 (−0.09–2.19	96.8 (64.1–98.7)	104.8 (83.3–108.2)	100.9 (78.9–105.5)
	*p* value *		0.186	0.088	**0.001**	0.168
RT Planning Technique	VMAT	109 (79.0)	0.06 (−0.49–4.07)	96.2 (66.8–99.1)	105.5 (81.5–108.8)	101.0 (77.7–105.5)
	IMRT	29 (21.0)	0.06 (−0.44–2.12)	96.4 (64.1–98.8)	104.8 (83.3–108.7)	100.7 (78.9–103.0)
	*p* value *		0.681	0.336	**0.020**	0.399
RT Field	Pelvis	24 (17.4)	0.07 (−0.44–1.9)	96.1 (71.6–98.8)	104.9 (81.5–108.7)	101.1 (77.7–103.0)
	Prostate	71 (51.4)	0.08 (−0.33–4.0)	96.5 (69.8–98.3)	105.3 (100.4–108.1)	101.0 (97.9–105.5)
	Prostate + SV	43 (31.2)	0.04 (−0.49–2.1)	96.0 (64.1–99.1)	105.6 (83.3–108.8)	100.9 (78.9–103.4)
	*p* value **		0.348	0.664	0.357	0.813
Total Dose (Gy)	70 ^1^	30 (21.7)	0.07 (−0.09–2.1)	97.4 (80.6–98.7)	105.2 (103.8–108.2)	101.1 (98.8–102.8)
	72.5 ^2^	19 (13.8)	1.0 (0–1.2)	95.2 (84.1–98.1)	104.1 (100.4–106.2)	100.5 (97.9–105.5)
	74 ^3^	1 (0.7)	0.09	80.0	107.4	101.1
	76 ^4^	24 (17.4)	0.14 (−0.33–4.0)	96.3 (69.8–99.1)	106.1 (102.4–108.8)	100.9 (98.4–102.9)
	78 ^5^	39 (28.3)	0.05 (−0.49–1.9)	95.8 (66.8–99.0)	105.8 (81.5–108.7)	101.1 (77.7–103.4)
	80 ^6^	25 (18.1)	0.06 (−0.05–1.7)	96.0 (64.1–98.2)	104.0 (83.3–106.8)	100.2 (78.9–102.2)
	*p* **		0.687	**0.016**	**<0.001**	**0.002**
	Post-hoc *			1 > 5, 6	4, 5 > 6	5 > 6
Prostate Volume (cc)	<50	35 (25.4)	0.06 (−0.10–1.84)	96.4 (66.8–98.8)	105.3 (81.5–108.7)	100.8 (77.7–103.4)
	50–70	46 (33.3)	0.07 (−0.23–2.12)	96.7 (76.1–99.0)	105.0 (102.5–107.9)	100.9 (98.7–102.8)
	70–100	32 (23.2)	0.14 (−0.49–4.07)	96.5 (81.3–98.3)	105.0 (101.4–108.2)	101.1 (98.4–101.7)
	>100	25 (18.1)	0.05 (−0.44–1.25)	95.6 (64.1–99.1)	105.8 (83.3–108.8)	101.1 (78.9–105.5)
	*p* **		0.512	0.385	0.905	0.709

*p* values were calculated using the Mann–Whitney U test for two-group comparisons (*) and the Kruskal–Wallis H test for comparisons involving more than two groups (**). Post hoc analyses were performed using the Dunn–Bonferroni correction where applicable. Superscript numbers (^1–6^) indicate predefined radiotherapy dose levels as follows: 70 Gy^1^, 72.5 Gy^2^, 74 Gy^3^, 76 Gy^4^, 78 Gy^5^, and 80 Gy^6^. Post-hoc comparisons are indicated by. Post-hoc pairwise comparisons (*) indicate statistically significant differences between radiotherapy dose groups. For example, “1 > 5, 6” denotes that dose group 1 differs significantly from dose groups 5 and 6. Bold values indicate statistically significant *p*-values. Abbreviations: PSA: prostate-specific antigen, RT: radiotherapy, PET: positron emission tomography, PSMA: prostate-specific membrane antigen, Conv: conventional, Hypo: hypofractionated, VMAT: volumetric modulated arc therapy, IMRT: intensity-modulated radiotherapy, SV: seminal vesicles, Min: minimum, Max: maximum.

**Table 3:** Dosimetric comparison of organs at risk according to PSMA PET fusion, intraprostatic boost, fractionation, planning technique, treatment volume, and total dose.

Characteristics		Rectum					Bladder			Femoral Heads			Penile Bulb	Body
		Min	Max	Mean	V65	V50	Min	Max	Mean	Min	Max	Mean	Mean	Max
		Median (Min–Max)	Median (Min–Max)	Median (Min–Max)	Median (Min–Max)	Median (Min–Max)	Median (Min–Max)	Median (Min–Max)	Median (Min–Max)	Median (Min–Max)	Median (Min–Max)	Median (Min–Max)	Median (Min–Max)	Median (Min–Max)
PSMA PET/CT Imaging	No	3.2 (1.2–13.2)	103.8 (95.7–107.4)	33.9 (19.0–46.3)	7.0 (0.7–19.8)	13.6 (6.3–21.9)	4.8 (0.9–24.4)	104.1 (99.1–108.2)	35.9 (14.8–66.7)	3.6 (0.3–105.0)	57.0 (29.6–69.8)	26.0 (10.9–56.2)	34.4 (11.6–64.3)	106.8 (102.4–109.5)
	Yes	2.9 (1.0–9.8)	103.5 (98.9–106.9)	32.6 (19.0–54.0)	5.2 (1.2–21.7)	11.4 (4.4–29.2)	3.7 (0.7–35.5)	103.7 (99.7–108.5)	36.9 (11.6–68.8)	1.2 (0–28.2)	55.0 (16.0–82.3)	21.4 (1.4–41.0)	34.3 (12.5–76.0)	105.9 (101.7–109.6)
	*p* value *	0.081	0.854	0.475	**0.007**	**0.027**	0.753	0.912	0.991	**0.002**	0.993	**0.004**	0.874	**<0.001**
Intraprostatic Focal Boost	No	3.1 (1.0–13.2)	103.8 (95.7–107.4)	33.7 (19.0–54.0)	5.7 (0.7–21.7)	12.8 (4.4–29.2)	3.9 (0.7–24.4)	104.5 (99.1–108.5)	37.4 (11.6–66.7)	1.5 (0.1–105.0)	56.5 (16.0–82.3)	23.3 (7.8–56.2)	34.7 (11.6–76.0)	106.6 (103.2–109.6)
	Yes	2.6 (1.0–6.8)	103.2 (98.9–106.7)	30.4 (20.6–51.4)	5.3 (2.9–16.5)	11.1 (7.3–26.0)	3.7 (1.1–35.5)	102.6 (99.7–107.9)	36.2 (13.4–68.8)	1.1 (0–26.2)	52.7 (34.7–74.3)	**20.5** (1.4–36.9)	34.3 (14.4–55.6)	104.7 (101.7–107.9)
	*p* value *	0.083	0.026	0.323	0.334	0.125	0.905	**0.001**	0.479	0.12	0.553	**0.007**	0.918	**<0.001**
Fractionation	Conv	3.6 (1.2–13.2)	103.8 (95.7–107.2)	34.4 (19.0–54.0)	6.7 (0.7–15.4)	13.7 (4.4–29.2)	4.8 (0.9–24.4)	103.5 (99.1–108.2)	37.5 (13.4–64.4)	2.5 (0–28.2)	58.0 (29.6–82.3)	25.0 (7.8–41.0)	35.1 (11.6–76.0)	106.3 (103.2–109.6)
	Hypo	2.6 (1.0–6.8)	103.5 (98.9–107.4)	30.3 (20.6–51.4)	4.9 (1.2–21.7)	11.1 (6.2–28.9)	3.6 (0.7–35.5)	103.8 (99.7–108.5)	34.2 (11.6–68.8)	1.1 (0.1–105.0)	53.0 (16.0–74.3)	21.1 (1.4–56.2)	34.1 (14.4–55.6)	106.4 (101.7–108.7)
	*p* value *	**0.001**	0.718	**0.021**	**<0.001**	**0.003**	0.116	0.361	0.483	**0.007**	**0.031**	**0.003**	0.338	0.12
RT Planning Technique	VMAT	3.0 (1.0–13.2)	103.7 (95.7–107.4)	31.7 (19.0–49.9)	5.5 (0.7–21.7)	12.2 (4.4–29.2)	3.6 (0.7–24.4)	104.2 (99.1–108.5)	34.2 (11.6–66.7)	1.5 (0–105.0)	54.5 (16.0–71.0)	22.4 (10.0–56.2)	34.1 (11.6–64.3)	106.5 (102.4–109.6)
	IMRT	4.4 (1.0–9.8)	103.4 (98.9–106.8)	39.6 (23.3–54.0)	5.3 (2.1–16.5)	13.6 (6.2–26.8)	14.6 (2.1–35.5)	102.9 (100.5–106.6)	47.4 (25.7–68.8)	1.3 (0.2–26.9)	63.2 (40.0–82.3)	25.0 (1.4–40.0)	38.2 (13.7–76.0)	105.1 (101.7–107.6)
	*p* value *	**0.002**	0.407	**<0.001**	0.927	0.2	**<0.001**	**0.016**	**<0.001**	0.917	**<0.001**	0.297	0.128	**0.001**
Radiotherapy Field	Pelvis ^1^	4.8 (2.8–9.8)	103.3 (99.6–106.8)	42.4 (29.4–54.0)	5.5 (2.9–15.4)	14.2 (7.7–29.2)	16.5 (1.5–24.4)	103.0 (100.2–106.6)	47.5 (23.4–61.3)	1.6 (0.3–28.2)	63.5 (52.5–82.3)	27.1 (15.1–41.0)	40.3 (13.7–76.0)	105.1 (103.2–107.6)
	Prostate ^2^	2.7 (1.0–8.1)	103.8 (99.9–107.49)	30.3 (19.0–44.2)	5.3 (1.2–21.7)	11.4 (4.4–28.9)	3.0 (0.7–17.6)	104.9 (100.4–108.2)	29.9 (11.6–66.7)	1.4 (0.3–105.0)	52.0 (16.0–69.2)	21.0 (7.8–56.2)	34.1 (12.5–64.3)	106.5 (102.4–108.8)
	SV ^3^	2.9 (1.3–13.2)	103.4 (95.7–106.9)	31.4 (20.6–51.49)	5.8 (0.7–16.5)	12.5 (6.2–26.0)	3.8 (1.0–35.5)	103.4 (99.1–108.5)	37.3 (14.0–68.8)	2.1 (0–26.2)	58.1 (29.6–73.9)	22.9 (1.4–39.1)	33.7 (11.6–61.9)	106.5 101.7 (109.6)
	*p* **	**<0.001**	0.109	**<0.001**	0.413	0.057	**<0.001**	**0.013**	**<0.001**	0.697	**<0.001**	**0.002**	0.138	**0.002**
	post-hoc *	1 > 2.3		1 > 2.3			1 > 2.3	2 > 1.3	1 > 2.3		1.3 > 2	1 > 2.3		2.3 > 1
Total Dose (Gy)	70 ^1^	2.6 (1.0–6.1)	103.8 (99.9–107.4)	30.0 (23.3–40.2)	4.0 (1.2–21.7)	10.9 (6.2–28.9)	3.7 (0.7–17.6)	105.0 (101.2–108.5)	37.1 (11.6–66.7)	1.0 (0.3–105.0)	53.4 (16.0–72.7)	21.0 (7.8–56.2)	32.3 (14.5–50.0)	106.6 (104.1–108.7)
	72.5 ^2^	2.3 (1.0–4.9)	103.4 (102.1–106.7)	28.8 (20.6–40.3)	5.1 (2.9–8.5)	10.7 (7.3–14.5)	2.8 (1.1–14.4)	103.7 (100.6–107.9)	29.2 (13.4–51.9)	1.5 (0.4–26.2)	48.5 (34.7–66.5)	17.6 (1.4–36.9)	34.3 (14.4–47.1)	105.0 (102.4–107.9)
	74 ^3^	2	107.2	29.7	7.1	13.5	1.7	107.1	14.8	1.4	45.8	19.9	17.7	107.4
	76 ^4^	3.0 (1.2–8.1)	104.2 (101.3–106.9)	32.9 (19.0–44.2)	7.1 (2.0–12.0)	13.9 (4.4–21.4)	3.5 (0.9–11.4)	105.4 (99.1–108.2)	35.6 (14.0–64.4)	3.6 (0.1–9.0)	56.9 (33.0–67.9)	26.3 (11.7–32.9)	30.6 (12.5–64.3)	107.3 (104.1–109.6)
	78 ^5^	4.1 (1.7–13.2)	103.3 (95.7–106.8)	36.5 (23.0–54.0)	6.3 (0.7–15.4)	13.7 (6.3–29.2)	5.4 (1.1–24.4)	103.0 (100.0–107.4)	41.6 (18.6–61.3)	2.4 (0.3–28.2)	59.1 (29.6–82.3)	25.6 (10.9–41.0)	38.2 (11.6–76.0)	106.1 (103.2–109.5)
	80 ^6^	3.6 (1.7–6.8)	102.8 (98.9–105.4)	34.0 (23.0–51.4)	5.7 (3.3–16.5)	12.6 (7.7–26.0)	6.6 (1.3–35.5)	102.4 (99.7–107.0)	38.2 (21.4–68.8)	1.1 (0–16.7)	60.9 (43.8–74.3)	22.2 (15.0–34.4)	34.3 (16.6–55.6)	104.4 (101.7–107.0)
	*p* **	**<0.001**	**0.039**	**0.004**	**0.001**	**0.008**	**0.002**	**<0.001**	**0.026**	**0.01**	**<0.001**	**<0.001**	0.213	**<0.001**
	Post hoc *	5.6 > 1.2	4.5 > 6	5 > 1.2	4.5 > 1	4.5 > 1	5.6 > 1.2.3.4	2.4 > 5.6	5 > 2.3	4.5 > 6	4.5.6 > 1.2.3	4.5 > 2.6		4 > 6

*p* values were calculated using the Mann–Whitney U test for two-group comparisons (*) and the Kruskal–Wallis H test for comparisons involving more than two groups (**). Superscript numbers (^1–6^) indicate predefined radiotherapy dose levels as follows: 70 Gy^1^, 72.5 Gy^2^, 74 Gy^3^, 76 Gy^4^, 78 Gy^5^, and 80 Gy^6^. Post hoc analyses were performed using the Dunn–Bonferroni correction where applicable. V65 and V50 represent the percentage of the rectum volume receiving ≥65 Gy and ≥50 Gy, respectively. Abbreviations: PSA: prostate-specific antigen, RT: radiotherapy, PET: positron emission tomography, PSMA: prostate-specific membrane antigen, Conv: conventional, Hypo: hypofractionated, VMAT: volumetric modulated arc therapy, IMRT: intensity-modulated radiotherapy, SV: seminal vesicles, Min: minimum, Max: maximum, and Mean: mean dose.

## Data Availability

The raw data in this study may be obtained from the corresponding author upon reasonable request.
